# Basil and Cinnamon Essential Oils Improve Oxidative Stability and Fatty Acid Composition of Vegetable Oil Blends During Deep-Frying

**DOI:** 10.3390/foods15132284

**Published:** 2026-06-25

**Authors:** Tran Thi Ly, Pham Thi Vinh, Ligang Yang, Guiju Sun

**Affiliations:** 1 Department of Nutrition and Food Hygiene, School of Public Health, Southeast University, Nanjing 210009, China; tranlytbs@gmail.com (T.T.L.); yangligang2012@163.com (L.Y.); 2 Institute of Biotechnology and Food Technology, Thai Nguyen University of Agriculture and Forestry, Thai Nguyen 250000, Vietnam; phamthivinh@tuaf.edu.vn

**Keywords:** basil essential oil, cinnamon essential oil, deep-frying, lipid oxidation, oxidative stability, fatty acid degradation

## Abstract

The present study evaluated the effects of basil essential oil (BEO) and cinnamon essential oil (CEO) on the oxidative stability and fatty acid degradation of vegetable oil blends during deep-frying. Three vegetable oil blends (OB1, OB2, and OB3), formulated with different n-6/n-3 fatty acid ratios, were supplemented with essential oils at concentrations of 200, 400, 800, and 1200 ppm and subjected to repeated deep-frying at 180 ± 5 °C for 8 h with periodic sampling. Changes in fatty acid composition, peroxide value (PV), acid value (AV), malondialdehyde (MDA), and *p*-anisidine value (AnV) were performed to characterize lipid oxidation under thermal stress. Prolonged frying significantly increased oxidation indices and accelerated the degradation of polyunsaturated fatty acids, particularly n-3 fatty acids, leading to an increased n-6/n-3 ratio. However, supplementation with basil and cinnamon essential oils effectively inhibited lipid oxidation and reduced fatty acid degradation compared with the control. Both essential oils decreased PV, AV, MDA, and AnV in a concentration-dependent manner, with more pronounced effects at 800 and 1200 ppm. Kinetic analysis showed that MDA formation followed a zero-order model, while PV changes fitted a first-order kinetic model, with R2 values ranging from 0.857 to 0.932. These findings suggest that basil and cinnamon essential oils enhance the oxidative stability of vegetable oil blends during deep-frying by reducing lipid oxidation and slowing unsaturated fatty acid degradation, highlighting their potential as natural antioxidants for frying oil applications.

## 1. Introduction

Oxidative stability is a critical factor determining the quality, safety, and shelf life of vegetable oils, particularly during high-temperature processing such as deep-frying. Under frying conditions, oils undergo complex degradation reactions including thermoxidation, polymerization, and hydrolysis, leading to the formation of primary and secondary oxidation products such as hydroperoxides, aldehydes, and ketones, which adversely affect sensory quality and nutritional value [1]. In addition, repeated exposure to high temperatures accelerates the depletion of natural antioxidants and promotes fat degradation, especially in oils rich in polyunsaturated fatty acids, thereby reducing overall oil stability [2].

In addition to oxidative stability, the fatty acid composition of edible oils, particularly the balance between n-6 and n-3 polyunsaturated fatty acids, is an important nutritional consideration. An imbalanced n-6/n-3 ratio has been associated with adverse health outcomes and reflects the quality of lipid sources in the diet [3,4]. From a technological perspective, oils with higher levels of polyunsaturated fatty acids, especially n-3 fatty acids, are more susceptible to oxidation during high-temperature processing such as deep-frying, leading to rapid degradation of nutritional quality. Therefore, designing oil systems with controlled n-6/n-3 ratios while maintaining oxidative stability represents a key challenge in the development of functional edible oils. During deep-frying, oils are subjected to high temperatures in the presence of oxygen and moisture, which accelerate free radical-mediated lipid oxidation processes and result in the rapid formation of primary and secondary oxidation products such as hydroperoxides, aldehydes, and other reactive carbonyl compounds [5]. These reactions not only deteriorate sensory attributes but also reduce the nutritional quality of oils and promote the formation of potentially toxic compounds, such as reactive aldehydes and polymerization products. Therefore, enhancing oxidative stability under deep-frying conditions remains a significant challenge in food lipid science [5,6].

In recent years, plant-derived essential oils have attracted increasing attention as natural antioxidants due to their potent bioactivity and their potential to replace synthetic additives, driven by growing consumer demand for clean-label and safer food products [7]. Among these, basil (*Ocimum basilicum* L.) and cinnamon (*Cinnamomum* spp.) essential oils are particularly promising, as they are rich in bioactive compounds such as phenylpropanoids, aromatic aldehydes, monoterpenes, and sesquiterpenes, which have been widely reported to exhibit antioxidant properties [8,9].

The chemical composition of basil and cinnamon essential oils has been widely reported to differ significantly, with basil oil typically rich in phenylpropanoids and cinnamon oil characterized by aromatic aldehydes. These compositional differences are expected to influence their antioxidant mechanisms and effectiveness, particularly under high-temperature conditions such as deep-frying [8].

Despite the well-documented antioxidant properties of plant essential oils, their performance in complex frying systems remains inadequately characterized. Deep-frying involves simultaneous thermo-oxidation, hydrolysis, and polymerization reactions, which can significantly alter lipid structures and accelerate fatty acid degradation [1,10]. Although essential oils have been shown to improve oxidative stability in model and real oil systems [5,11], limited information is available regarding their effectiveness in preserving polyunsaturated fatty acids and modulating the formation of primary and secondary oxidation products during prolonged frying processes. Therefore, a comprehensive evaluation under realistic frying conditions is still needed.

Therefore, the present study aims to investigate the effects of basil and cinnamon essential oils on the oxidative stability and fatty acid composition of vegetable oil blends during deep-frying. Changes in key quality indicators, including peroxide value (PV), acid value (AV), *p*-anisidine value (AnV), thiobarbituric acid reactive substances, and fatty acid composition, were systematically evaluated at different essential oil concentrations. The findings are expected to provide insight into the role of essential oil composition in modulating lipid oxidation under frying conditions and to support their application as natural antioxidants in edible oil systems.

However, limited studies have compared the effects of different essential oils in vegetable oil blends with distinct fatty acid profiles during deep-frying. In particular, information on the comparative effects of basil and cinnamon essential oils on oxidative stability and n-3/n-6 fatty acid preservation remains scarce. Therefore, this study addresses this gap by evaluating their effects on oxidation parameters and fatty acid composition in blended oils during deep-frying.

## 2. Materials and Methods

### 2.1. Preparation of Oil Blends

Three oil blends (OB1, OB2, and OB3) were formulated using sunflower oil (SFO), flaxseed oil (FSO), olive oil (OVO), and palm kernel oil (PKO), which were obtained from the Malaysian Palm Oil Board (MPOB, Kuala Lumpur, Malaysia). The blend compositions were generated using a MATLAB-based multi-objective linear programming model (MATLAB R2023b, MathWorks, Natick, MA, USA) developed to achieve nutritionally balanced fatty acid profiles. The optimization was performed to obtain a target SFA:MUFA:PUFA ratio of 1:1:1, where SFA, MUFA, and PUFA represent saturated, monounsaturated, and polyunsaturated fatty acids, respectively, while producing blends with different n-6/n-3 ratios. Based on the model output, three blends with initial n-6/n-3 ratios of approximately 1, 3, and 5 were selected for subsequent frying experiments and designated as OB1, OB2, and OB3, respectively.

The compositions of the blends were as follows:

OB1: 17.37% SFO, 28.84% FSO, 22.67% OVO, 31.12% PKO;

OB2: 35.11% SFO, 14.30% FSO, 20.75% OVO, 29.84% PKO;

OB3: 41.02% SFO, 9.45% FSO, 20.11% OVO, 29.42% PKO.

SFO, FSO, OVO, and PKO oil were purchased from commercial suppliers (food-grade quality, China) and stored under dark and refrigerated conditions (4 °C) prior to use. The fatty acid compositions of the individual oils used for blend formulation are presented in Supplementary Table S1.

The initial n-6/n-3 ratios of OB1, OB2, and OB3 were approximately 1.0, 3.0, and 5.0, respectively. The initial quality of the oils complied with edible oil standards. The oil blends were prepared on a weight/weight (*w*/*w*) basis to ensure accurate formulation of fatty acid composition.

### 2.2. Research Design

Basil essential oil and cinnamon essential oil were added to each oil blend at concentrations of 0 (control), 200, 400, 800, and 1200 ppm. All samples (OB1, OB2, and OB3) were subjected to identical deep-frying conditions to investigate the concentration-dependent effects of essential oils on oxidative stability and fatty acid degradation.

Basil essential oil was extracted from fresh basil leaves (*Ocimum basilicum* L.), whereas cinnamon essential oil was obtained from dried cinnamon bark (*Cinnamomum cassia* Presl.). The botanical identity of the plant materials was confirmed prior to extraction. Essential oils were extracted in-house using steam distillation (Clevenger-type apparatus) and stored in sealed amber vials at 4–5 °C until analysis.

The essential oils used in this study have been previously characterized based on their major volatile constituents, with basil essential oil reported to be rich in estragole, while cinnamon essential oil is dominated by cinnamaldehyde.

### 2.3. Frying Procedure and Sample Collection

Deep-frying experiments were performed using a temperature-controlled electric deep fryer (Shanghai Yixi Food Machinery Co., Ltd., Shanghai, China). The oil temperature was continuously monitored using a digital thermostat (Xuzhou Sanhe Automatic Control Equipment Co., Ltd., Xuzhou, Jiangsu, China) and verified with a calibrated thermocouple probe (±0.5 °C accuracy; Testo SE & Co. KGaA, Titisee-Neustadt, Germany). Each frying system contained 3 L of oil and was operated without oil replenishment throughout the experiment.

The frying process followed a repeated batch cycle under controlled conditions. In each cycle, 500 g of French fries were fried at 180 ± 5 °C for 4.5 min. The French fries were used in frozen form (industrial pre-fried potato strips stored at −18 °C) and were introduced directly into the fryer without thawing. The introduction of frozen product resulted in a temporary decrease in oil temperature after each loading; however, the thermostatic control system ensured that the temperature rapidly returned to 180 ± 5 °C before the next stage of frying. After each frying batch, the oil was maintained at the same temperature for a holding period of 25.5 min before the next batch was introduced. After thermal recovery, this intermittent holding step allowed the system to stabilize before the next frying cycle. This intermittent frying regime was applied continuously to simulate realistic frying practices and to ensure sustained thermal and oxidative stress on the oil.

The total duration of the frying experiment was 8 h. Oil samples (50 g) were collected at 2, 4, 6 and 8 h to monitor changes in oxidative stability and fatty acid composition. Immediately after collection, the samples were transferred into screw-cap vials and stored at −20 °C until further analysis.

All experiments were conducted in triplicate for each oil system to ensure reproducibility and reliability of the results. Although a relatively high product-to-oil ratio was used (500 g per 3 L oil), this condition was intentionally selected to simulate industrial batch frying and to impose realistic thermal and oxidative stress on the oil system. Commercial frozen French fries (industrial pre-fried potato strips) were used as the food matrix in this study. The samples were purchased from a local commercial market in China and stored at −18 °C prior to frying. They were used directly without thawing or any additional processing. Due to the commercial nature of the product, detailed information regarding industrial pre-frying conditions (including oil type and processing parameters) was not available.

To evaluate the oxidative stability and fatty acid degradation of vegetable oil blends during deep-frying, fatty acid composition and oxidation-related parameters were analyzed.

### 2.4. Fatty Acid Composition and Oxidative Stability Analysis

Fatty acid composition was determined by gas chromatography (GC) after conversion to fatty acid methyl esters (FAMEs) according to [12]. Briefly, 2 g of oil sample was saponified with 40 mL of 2% NaOH in methanol under a nitrogen atmosphere (99.99%) at 80 °C for 30 min. Fatty acids were subsequently methylated using 4 mL of 15% boron trifluoride (BF_3_) in methanol to obtain FAMEs.

FAMEs were analyzed using a gas chromatograph (PU 4410, Philips, Cambridge, UK) equipped with an HP-Innowax capillary column (30 m × 0.25 mm × 0.50 μm; Agilent Technologies, Santa Clara, CA, USA) and a flame ionization detector (FID). Nitrogen (99.99%) was used as the carrier gas at a flow rate of 1.0 mL/min. The oven temperature was programmed from 140 °C to 240 °C at a rate of 4 °C /min, with an initial hold of 5 min at 140 °C and a final hold of 15 min at 240 °C. Samples (1 μL) were injected using a split ratio of 100:1. The injector and detector temperatures were maintained at 250 °C. Fatty acids were identified by comparing retention times with those of FAME reference standards analyzed under identical chromatographic conditions. Fatty acid composition was quantified by peak-area normalization and expressed as percentage of total fatty acid methyl esters (% total FAMEs).

PV, AV, and AnV were determined according to ISO 3960:2007, ISO 660:2009, and ISO 6885:2006, respectively [13,14,15]. For PV determination, approximately 5 g of oil was dissolved in a chloroform–acetic acid mixture, reacted with potassium iodide, and titrated with standardized sodium thiosulfate solution. AV was determined by titration of oil dissolved in an ethanol-diethyl ether mixture with 0.1 N KOH solution using phenolphthalein as an indicator. For AnV determination, oil samples were dissolved in isooctane and reacted with *p*-anisidine reagent, and absorbance was measured at 350 nm. Results were calculated according to the corresponding ISO methods and expressed as meq O_2_/kg oil (PV), mg KOH/g oil (AV), and anisidine units (AnV).

Malondialdehyde (MDA) content was measured according to GB 5009.168-2016 with slight modifications using the thiobarbituric acid reactive substances (TBARS) assay [12]. Specifically, 5 g of oil sample was mixed with 50 mL of trichloroacetic acid solution containing EDTA and shaken at 50 °C for 30 min. After filtration, 5 mL of filtrate was reacted with 5 mL of thiobarbituric acid solution (2.88 g/L) at 90 °C for 30 min. After cooling, absorbance was measured at 532 nm using a UV-Vis spectrophotometer (UV-1800, Shimadzu Corporation, Kyoto, Japan). Results were calculated from a calibration curve (R^2^ = 0.9985) and expressed as mg MDA equivalents/kg oil. All analyses were conducted in triplicate.

### 2.5. Kinetic Modeling of Lipid Oxidation During Deep-Frying

The kinetic change of lipid oxidation during deep-frying was evaluated using PV and MDA as representative indicators of primary and secondary oxidation, respectively. Kinetic analysis was performed using the oxidation data obtained from untreated oil blends (OB1-OB3) during heating treatment.

The kinetic equations used were:

$$ln(PV_{t})=ln(PV_{0})+kt\;\mathrm{and}\;MDA_{t}=MDA_{0}+kt$$

where PV_t_ and MDA_t_ represent the oxidation indices at frying time t, PV_0_ and MDA_0_ are the initial values, and k is the apparent oxidation rate constant. Linear regression analysis and kinetic fitting were performed using OriginPro software (version 2026, OriginLab Corporation, Northampton, MA, USA). Model suitability was evaluated based on the coefficient of determination R^2^, the statistical significance of the regression model (*p*-value), and the root mean square error (RMSE). A regression model was considered statistically significant when *p* < 0.05. The apparent oxidation rate constant (k) was obtained from the fitted kinetic equations and used to characterize the oxidation rate of the oil samples. The RMSE was used to evaluate the deviation between experimental and predicted values, with lower RMSE values indicating better agreement and model fitting. PV changes were best described by the first-order kinetic model, whereas MDA formation followed zero-order kinetics.

### 2.6. Statistical Analysis

All experiments were conducted in triplicate, and results were expressed as mean ± standard deviation (SD), with *p* < 0.05 indicating statistical significance. Statistical differences among treatments were evaluated using analysis of variance (ANOVA), followed by Bonferroni-adjusted post hoc comparisons to determine significant differences between mean values.

## 3. Results and Discussion

### 3.1. Effects of Essential Oils on Fatty Acid Composition of Oil Blends During Deep-Frying

The changes in fatty acid composition of oil blends during deep-frying in the presence of basil and cinnamon essential oils are presented in Table 1, Table 2, Table 3 and Table 4.

Overall, both n-6 and n-3 fatty acids gradually decreased with increasing frying time, indicating progressive lipid degradation under thermal oxidation conditions. However, n-3 fatty acids consistently exhibited a faster rate of decline compared to n-6 fatty acids, resulting in a continuous increase in the n-6/n-3 ratio during frying. This difference can be explained by the higher susceptibility of polyunsaturated fatty acids to oxidative degradation. In particular, linolenic acid (C18:3), which contains three double bonds, is more susceptible to hydrogen abstraction at bis-allylic positions and subsequent peroxidation than linoleic acid (C18:2), which contains two double bonds [16]. In addition, heat and mass transfer phenomena during deep-frying further accelerate lipid oxidation by continuously exposing fresh oil surfaces to oxygen and moisture, intensifying hydroperoxide formation and decomposition reactions [17,18]. Therefore, oils rich in n-3 fatty acids are inherently less stable under repeated frying conditions compared to n-6 fatty acids, as also reported in recent reviews on frying oil degradation mechanisms [1,2]. This observation can be further interpreted in relation to the initial fatty acid profiles of the oil blends, particularly their PUFA content. Recent nutritional studies have highlighted that not only total PUFA levels but also the balance between omega-6 and omega-3 fatty acids is critical for assessing oil nutritional quality and oxidation susceptibility [3,4]. Oils with higher PUFA proportions tend to undergo faster lipid peroxidation due to increased radical formation potential, especially under high-temperature frying conditions [6]. Therefore, the initial fatty acid profile likely influenced the oxidative stability and degradation pattern of each oil blend during frying.

The effect of basil essential oil on n-6 fatty acid retention during frying is presented in Table 1.

As shown in Table 1, the addition of basil and cinnamon essential oils improved the oxidative stability of fatty acids during frying. In general, all treated oil blends showed a slower reduction in both n-6 and n-3 fatty acids compared to the control. This effect became more evident with increasing essential oil concentration, particularly at 800 and 1200 ppm, while only minor improvements were observed at 200 ppm. After 8 h of frying, higher retention of n-6 fatty acids was observed in all essential oil-treated samples compared to the control. A similar trend was also observed for n-3 fatty acids, although their contents still decreased markedly due to their higher susceptibility to thermal oxidation under frying conditions. In addition to n-6 fatty acids, the effect of basil essential oil on n-3 fatty acid stability during deep-frying is shown in Table 2.

As presented in Table 2, n-3 fatty acids decreased markedly during frying in all oil blends due to their high susceptibility to thermal oxidation. Nevertheless, basil essential oil supplementation significantly reduced the degradation rate of n-3 fatty acids compared with the control samples. The protective effect was concentration-dependent, with 800 and 1200 ppm showing the greatest retention of linolenic acid after prolonged frying. However, despite the antioxidant protection provided by basil essential oil, substantial losses of n-3 fatty acids were still observed after 8 h of frying, especially in OB3 samples.

Similar protective effects were also observed in oil blends supplemented with cinnamon essential oil. The changes in n-6 fatty acid content in cinnamon essential oil-treated oil blends are presented in Table 3.

As shown in Table 3, cinnamon essential oil effectively improved the retention of n-6 fatty acids during frying compared with the control. Increasing concentrations of cinnamon essential oil generally resulted in higher preservation of linoleic acid content, particularly at 800 and 1200 ppm. Among the tested oil blends, OB3 exhibited the highest n-6 fatty acid retention after prolonged frying in the presence of cinnamon essential oil.

The corresponding changes in n-3 fatty acid content during frying are shown in Table 4.

According to Table 4, cinnamon essential oil also delayed the degradation of n-3 fatty acids during deep-frying. However, n-3 fatty acids still exhibited a substantially faster decline than n-6 fatty acids because of their greater oxidative instability. A pronounced reduction in n-3 fatty acid content was observed in control samples after prolonged frying, whereas essential oil-treated samples maintained relatively higher levels, particularly at 800 and 1200 ppm.

The calculated n-6/n-3 ratios (Supplementary Table S2) increased significantly in all samples during frying, especially in OB3, where a sharp rise was observed in the control group. This was mainly attributed to the near depletion of n-3 fatty acids, resulting in a “ratio amplification effect” when the denominator becomes extremely low. Therefore, the markedly high n-6/n-3 ratios observed in some OB3 samples should be interpreted cautiously, as they were largely caused by near depletion of n-3 fatty acids during prolonged frying. From a nutritional perspective, this indicates a deterioration in lipid quality, as the nutritional value of vegetable oils depends not only on total PUFA content but also on a balanced n-6/n-3 ratio.

The antioxidant activity of basil and cinnamon essential oils may be related to bioactive compounds such as estragole and cinnamaldehyde, which can act as radical scavengers and interrupt lipid oxidation chain reactions. Recent evidence has demonstrated that essential oils exert their antioxidant effects through multiple mechanisms, including radical scavenging, metal chelation, and inhibition of lipid hydroperoxide decomposition [19,20,21]. Their lipophilic nature further facilitates distribution within the oil phase, enhancing oxidative stability under high-temperature conditions. Lipid oxidation in edible oils generally involves free radical chain reactions associated with polyunsaturated fatty acids, and the formation of oxidation products significantly affects both nutritional and functional properties of frying oils [22]. Consistent with previous studies, essential oils have been widely reported as natural antioxidants capable of delaying lipid oxidation in both model and real food systems under thermal processing conditions [7,8,10].

However, prolonged deep-frying still resulted in unavoidable changes in fatty acid composition and the n-6/n-3 ratio, even in the presence of essential oils. This agrees with previous studies indicating that natural antioxidants can reduce lipid oxidation but cannot fully prevent degradation under continuous thermal stress [23,24,25]. Moreover, fatty acid changes during frying may also be influenced by mass transfer between food and oil systems, which can affect the apparent impact of antioxidant additives [26].

The effect of essential oils was concentration-dependent, with 800 and 1200 ppm providing the most effective protection against fatty acid degradation. However, no significant difference was observed between these two concentrations (*p* > 0.05), suggesting a saturation effect, whereby increasing the concentration beyond 800 ppm provided limited additional protective effects. Similar concentration-dependent effects with a plateauing effect at higher concentrations have been previously reported in frying systems, where excessive antioxidant addition does not proportionally enhance oxidative stability due to solubility limits and reaction kinetics constraints in bulk oil systems [23,27].

Overall, these results demonstrate that basil and cinnamon essential oils can effectively delay the degradation of unsaturated fatty acids and moderate the increase in the n-6/n-3 ratio during deep-frying, although some degree of lipid oxidation remained inevitable under prolonged heating conditions.

### 3.2. Effects of Essential Oils on Oxidative Stability of Oil Blends During Deep-Frying

The oxidative stability of OB1, OB2, and OB3 during repeated deep frying is presented in Table 5.

Overall, PV, AV, MDA, and AnV increased with frying time, reflecting the continuous progression of lipid oxidation under prolonged heating conditions [24,28]. The simultaneous increase in oxidation indices indicates sequential lipid oxidation, in which hydroperoxides formed during primary oxidation subsequently decomposed into secondary aldehydic compounds during prolonged heating [6].

**Figure 1:**
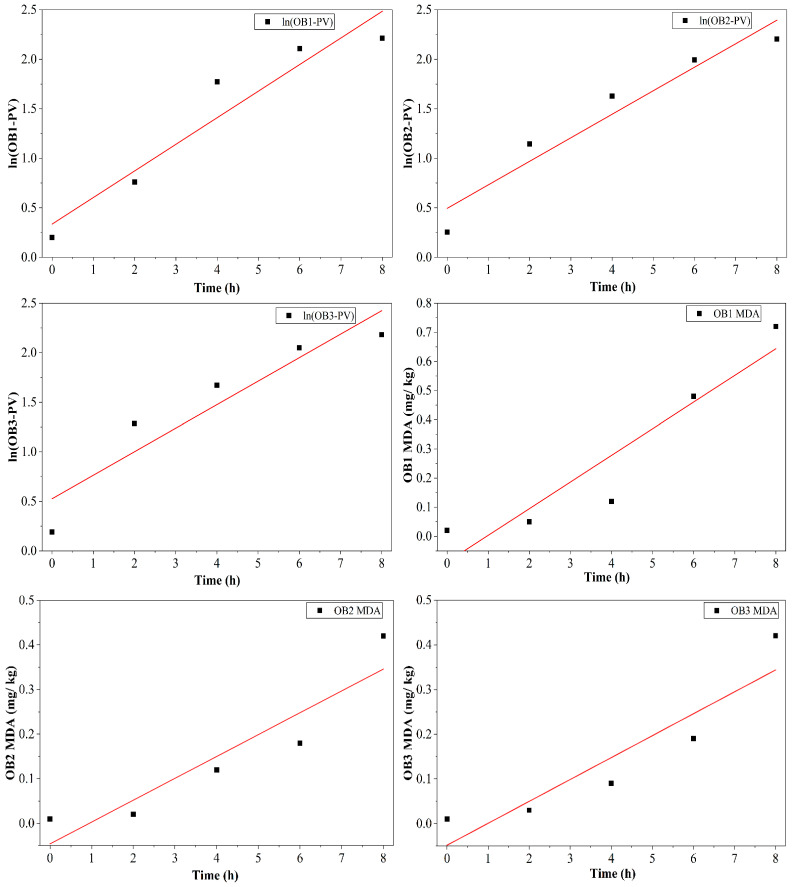
Zero-order (MDA formation) and first-order (PV changes) kinetic plots during heating treatment.

To further characterize lipid oxidation during heating, kinetic modeling was conducted using PV and MDA as representative indicators of primary and secondary oxidation, respectively. Although AV and AnV also increased during deep-frying, PV mainly reflects hydroperoxide formation during the early stages of oxidation, whereas MDA indicates the accumulation of secondary aldehydic compounds generated during prolonged heating [27,29]. The kinetic parameters of lipid oxidation during heating are presented in Table 6 and Figure 1.

MDA formation in the OB samples followed a relatively linear zero-order kinetic model, with R^2^ values ranging from 0.857 to 0.882. In contrast, PV changes were more suitably described by a first-order kinetic model, as indicated by the higher coefficients of determination (R^2^ = 0.882–0.932). All regression models were statistically significant (*p* < 0.05), confirming the suitability of the selected kinetic models for describing lipid oxidation during heating. Furthermore, the relatively low RMSE values demonstrated good agreement between the experimental and predicted data, indicating the reliability and predictive capability of the proposed models. These results suggest that the selected kinetic models were adequate for describing lipid oxidation during heating, which is consistent with previous studies on lipid oxidation kinetics reported by Farhoosh (2020) and Li et al. (2020) [27,29]. Among the tested samples, OB1 exhibited slightly higher oxidation rate constants for both MDA formation (k = 0.0915) and PV changes (k = 0.2688), indicating relatively lower oxidative stability during heating. The differences in k values among the oil blends may be related to variations in fatty acid composition and the stability of endogenous antioxidant compounds. Oil blends with higher oxidative susceptibility due to a higher content of unsaturated fatty acids may show increased hydroperoxide formation and generation of secondary oxidation products during heating. Moreover, thermal degradation of naturally occurring antioxidants during prolonged heating could reduce the antioxidant protection of the oil matrix, contributing to the higher oxidation rate observed in OB1. Overall, the high R^2^ values, statistically significant *p*-values (*p* < 0.05), and low RMSE values support the suitability of the selected kinetic models for describing the oxidation behavior of the oil blends during thermal treatment.

Across all oil systems, supplementation with basil and cinnamon essential oils significantly reduced (*p* < 0.05) the increase in oxidation indices compared with the control, indicating their role in limiting lipid oxidation under prolonged heating conditions. The antioxidant effect became more pronounced at concentrations of 800–1200 ppm. However, since no significant difference was observed between 800 and 1200 ppm in most oxidation indices, increasing the concentration beyond 800 ppm provided limited additional antioxidant benefit under the tested conditions.

The gradual increase in PV during frying indicates the formation and accumulation of lipid hydroperoxides under high-temperature conditions. However, oils supplemented with basil and cinnamon essential oils exhibited significantly lower PV values than the control, particularly during prolonged frying, suggesting their ability to delay lipid oxidation and limit the accumulation of early oxidation products. The effects of essential oils on PV are presented in Table 7.

The increase in AV during frying reflects triglyceride hydrolysis and free fatty acid formation caused by thermal and oxidative degradation [6,24]. Essential oil-treated samples consistently showed lower AV values than untreated oils, suggesting improved resistance to lipid degradation during prolonged heating. Since free fatty acids are more susceptible to oxidation than triglycerides, limiting their formation may also contribute to improved oxidative stability during frying [6].

The effects of essential oils on AV are summarized in Table 8.

MDA and AnV increased significantly during extended frying, particularly after 6 h. This trend suggests the progressive decomposition of lipid hydroperoxides into secondary oxidation products such as aldehydes and ketones during prolonged heating, as previously described in frying oil oxidation studies [1,25].

However, oils supplemented with basil and cinnamon essential oils showed markedly lower MDA accumulation than the control, especially at higher concentrations. This finding suggests that essential oils effectively inhibited secondary lipid oxidation during repeated frying cycles. The effects of essential oils on MDA formation are presented in Table 9.

AnV also increased significantly during frying, confirming the accumulation of secondary oxidation products such as aldehydes and ketones during prolonged thermal exposure [6,25]. Compared with the control, essential oil-treated samples exhibited significantly lower AnV values, indicating delayed decomposition of hydroperoxides into secondary aldehydic compounds. The effects of essential oils on AnV are shown in Table 10.

These findings are consistent with previous reports indicating that plant-derived essential oils can retard the degradation of polyunsaturated fatty acids during frying and improve the oxidative stability of oils during repeated heating cycles [24,25]. The observed protective effects may be attributed to the antioxidant compounds present in basil and cinnamon essential oils, which help limit the propagation of lipid oxidation reactions [21,25,30].

Regarding cinnamon essential oil, its strong antioxidant activity is mainly associated with cinnamaldehyde, which contributes to its ability to inhibit lipid oxidation. Previous studies have also demonstrated its effectiveness in food preservation systems due to both antimicrobial and antioxidant properties [20]. Similarly, the antioxidant activity of basil essential oil has been associated with bioactive compounds such as estragole and linalool, which are recognized as major constituents contributing to radical scavenging activity [31].

However, some studies have reported variable effects of essential oils on lipid oxidation products. Erol et al. (2022) noted that PV, AV, and other oxidation indices may not always clearly reflect antioxidant effects due to the instability of these compounds and their tendency to further react or decompose into secondary products [26]. Differences in experimental systems, such as fish-based frying substrates, may influence oxidation behavior due to mass transfer between food and oil phases [23]. Similar effects of matrix-driven oil degradation during frying have been reported in thermal oxidation studies of frying systems [1]. In addition, lipid oxidation pathways influenced by mass transfer and thermal degradation have been widely described in mechanistic studies [6].

In the present study, cinnamon essential oil generally exhibited slightly stronger protective effects than basil essential oil, particularly at later frying stages. This is in agreement with studies reporting that cinnamaldehyde contributes significantly to the antioxidant activity of cinnamon oil, although its effectiveness may vary depending on processing conditions and matrix interactions [24,28,30]. The stronger inhibition of MDA and AnV compared with PV suggests that essential oils were particularly effective at limiting hydroperoxide decomposition and secondary aldehyde formation rather than completely preventing oxidation initiation, as previously described in lipid oxidation studies [6,25,30].

In this study, lipid oxidation during deep frying followed a typical free radical chain mechanism governed by frying temperature, fatty acid unsaturation level, frying duration, antioxidant concentration, and coupled heat and mass transfer processes occurring between the food and oil phases, as previously reported in frying systems [17,18]. Essential oils do not alter the fundamental reaction pathway but primarily act by reducing the overall oxidation reaction rate through radical scavenging activity and hydroperoxide decomposition [28,30]. Consequently, basil and cinnamon essential oils delayed the progression of lipid oxidation during repeated frying cycles. However, prolonged frying subjects the oil to continuous thermal and oxidative stress, together with mass transfer interactions between food and oil, which gradually overwhelm the antioxidant protection and lead to unavoidable lipid degradation at extended frying times [17,25]. The use of frozen French fries and relatively high batch loading (500 g per 3 L oil) resulted in a transient decrease in oil temperature during each frying cycle; however, the temperature rapidly returned to the set point due to the thermostatic control system. This condition is representative of industrial batch frying practices.

## 4. Conclusions

This study demonstrated that basil and cinnamon essential oils effectively improved the oxidative stability of vegetable oil blends during repeated deep-frying. The results showed that n-3 fatty acids were more susceptible to thermal oxidation than n-6 fatty acids, resulting in an increased n-6/n-3 ratio during frying. However, essential oil supplementation reduced fatty acid degradation and attenuated the increase in oxidation indices (PV, AV, MDA, and AnV). The protective effects of basil and cinnamon essential oils were concentration-dependent, with more evident improvements observed at 800 and 1200 ppm. Kinetic analysis further confirmed that lipid oxidation followed predictable reaction models, with PV fitting a first-order model and MDA following a zero-order model. Overall, basil and cinnamon essential oils showed potential as natural antioxidants for improving oil stability under high-temperature frying conditions. However, this study is limited to thermal oxidation during repeated deep-frying. Further studies are required to evaluate their performance during long-term storage and their effects on sensory quality and shelf life.

## Figures and Tables

**Table 1:** Changes in n-6 fatty acid content of vegetable oil blends during deep-frying with basil essential oil.

Oil Blend	Time	0 ppm	200 ppm	400 ppm	800 ppm	1200 ppm
OB1	0 h	16.66 ± 0.01 ^aA^	16.66 ± 0.01 ^aA^	16.66 ± 0.01 ^aA^	16.66 ± 0.01 ^aA^	16.66 ± 0.01 ^aA^
	2 h	16.61 ± 0.01 ^aA^	16.62 ± 0.02 ^aA^	16.63 ± 0.02 ^aA^	16.61 ± 0.02 ^aA^	16.69 ± 0.03 ^aA^
	4 h	15.06 ± 0.07 ^bB^	15.08 ± 0.07 ^bB^	15.06 ± 0.02 ^bB^	15.84 ± 0.03 ^bA^	15.90 ± 0.01 ^bA^
	8 h	13.65 ± 0.05 ^cB^	13.63 ± 0.02 ^cB^	13.50 ± 0.03 ^cB^	14.50 ± 0.03 ^cA^	14.54 ± 0.03 ^cA^
OB2	0 h	24.92 ± 0.01 ^aA^	24.92 ± 0.01 ^aA^	24.92 ± 0.01 ^aA^	24.92 ± 0.01 ^aA^	24.92 ± 0.01 ^aA^
	2 h	24.75 ± 0.03 ^aA^	24.77 ± 0.02 ^aA^	24.66 ± 0.02 ^aA^	24.81 ± 0.01 ^aA^	24.87 ± 0.01 ^aA^
	4 h	23.50 ± 0.01 ^bB^	23.48 ± 0.02 ^bB^	23.38 ± 0.01 ^bB^	23.83 ± 0.02 ^bA^	23.87 ± 0.02 ^bA^
	8 h	21.95 ± 0.05 ^cA^	20.95 ± 0.02 ^cA^	20.78 ± 0.02 ^cB^	21.73 ± 0.03 ^cA^	21.81 ± 0.03 ^cA^
OB3	0 h	27.68 ± 0.01 ^aA^	27.68 ± 0.01 ^aA^	27.68 ±0.01 ^aA^	27.68 ± 0.01 ^aA^	27.68 ± 0.01 ^aA^
	2 h	27.67 ± 0.02 ^aA^	27.61 ± 0.03 ^aA^	27.65 ± 0.03 ^aA^	27.64 ± 0.02 ^aA^	27.68 ± 0.02 ^aA^
	4 h	25.81 ± 0.05 ^bB^	25.79 ± 0.03 ^bB^	25.70 ± 0.02 ^bB^	26.49 ± 0.03 ^bA^	26.62 ± 0.02 ^bA^
	8 h	24.26 ± 0.03 ^cC^	24.17 ± 0.03 ^cC^	24.01 ± 0.03 ^cB^	25.72 ± 0.04 ^cA^	25.82 ± 0.04 ^cA^

Values are expressed as mean ± standard deviation (*n* = 3) and reported as % total FAMEs. Different lowercase superscript letters indicate significant differences among frying times within the same oil blend and essential oil concentration. Different uppercase superscript letters indicate significant differences among essential oil concentrations at the same frying time within the same oil blend (two-way ANOVA followed by Tukey’s post hoc test, *p* < 0.05). 0 h: unheated oil before frying (initial state prior to thermal treatment).

**Table 2:** Changes in n-3 fatty acid content of vegetable oil blends during deep-frying with basil essential oil.

Oil Blend	Time	0 ppm	200 ppm	400 ppm	800 ppm	1200 ppm
OB1	0 h	16.64 ± 0.01 ^aA^	16.64 ± 0.01 ^aA^	16.64 ± 0.01 ^aA^	16.64 ± 0.01 ^aA^	16.64 ± 0.01 ^aA^
	2 h	16.58 ± 0.06 ^aA^	16.59 ± 0.03 ^aA^	16.51 ± 0.03 ^aA^	16.52 ± 0.03 ^aA^	16.59 ± 0.03 ^aA^
	4 h	14.12 ± 0.03 ^bA^	14.16 ± 0.05 ^bA^	14.08 ± 0.02 ^bA^	14.14 ± 0.03 ^bA^	14.05 ± 0.02 ^bA^
	8 h	10.17 ± 0.04 ^cB^	10.21 ± 0.03 ^cB^	10.12 ± 0.03 ^cB^	12.16 ± 0.04 ^cA^	12.23 ± 0.04 ^cA^
OB2	0 h	8.42 ± 0.01 ^aA^	8.42 ± 0.01 ^aA^	8.42 ± 0.01 ^aA^	8.42 ± 0.01 ^aA^	8.42 ± 0.01 ^aA^
	2 h	8.42 ± 0.04 ^aA^	8.38 ± 0.03 ^aA^	8.39 ± 0.03 ^aA^	8.34 ± 0.02 ^aA^	8.35 ± 0.02 ^aA^
	4 h	6.27 ± 0.03 ^bA^	6.29 ± 0.02 ^bA^	6.30 ± 0.01 ^bA^	6.33 ± 0.03 ^bA^	6.36 ± 0.02 ^bA^
	8 h	3.12 ± 0.04 ^cC^	3.18 ± 0.03 ^cC^	3.31 ± 0.03 ^cB^	5.22 ± 0.04 ^cA^	5.34 ± 0.04 ^cA^
OB3	0 h	5.52 ± 0.01 ^cA^	5.52 ± 0.01 ^cA^	5.52 ± 0.01 ^cA^	5.52 ± 0.01 ^cA^	5.52 ± 0.01 ^cA^
	2 h	5.47 ± 0.02 ^cA^	5.48 ± 0.03 ^cA^	5.48 ± 0.03 ^aA^	5.49 ± 0.02 ^cA^	5.53 ± 0.02 ^aA^
	4 h	2.31 ± 0.04 ^bA^	2.35 ± 0.03 ^bA^	2.46 ± 0.02 ^bA^	2.50 ± 0.03 ^bA^	2.49 ± 0.02 ^bA^
	8 h	0.89 ± 0.03 ^cB^	0.90 ± 0.03 ^cB^	0.11 ± 0.03 ^cB^	1.95 ± 0.04 ^cA^	1.99 ± 0.04 ^cA^

Values are expressed as mean ± standard deviation (*n* = 3) and reported as % total FAMEs. Different lowercase superscript letters indicate significant differences among frying times within the same oil blend and essential oil concentration. Different uppercase superscript letters indicate significant differences among essential oil concentrations at the same frying time within the same oil blend (two-way ANOVA followed by Tukey’s post hoc test, *p* < 0.05). 0 h: unheated oil before frying (initial state prior to thermal treatment).

**Table 3:** Changes in n-6 fatty acid content of vegetable oil blends during deep-frying with cinnamon essential oil.

Oil Blend	Time	0 ppm	200 ppm	400 ppm	800 ppm	1200 ppm
OB1	0 h	16.65 ± 0.02 ^aA^	16.65 ± 0.02 ^aA^	16.65 ± 0.02 ^aA^	16.65 ± 0.02 ^aA^	16.65 ± 0.02 ^aA^
	2 h	16.05 ± 0.02 ^aA^	16.09 ± 0.03 ^aA^	16.62 ± 0.03 ^aA^	16.63 ± 0.02 ^aA^	16.56 ± 0.02 ^aA^
	4 h	14.92 ± 0.02 ^bA^	14.98 ± 0.03 ^bA^	15.01 ± 0.02 ^bA^	15.01 ± 0.03 ^bB^	15.03 ± 0.02 ^bA^
	8 h	13.40 ± 0.02 ^cB^	13.44 ± 0.03 ^cB^	13.47 ± 0.03 ^cB^	14.52 ± 0.04 ^cA^	14.55 ± 0.04 ^cA^
OB2	0 h	24.92 ± 0.01 ^aA^	24.92 ± 0.01 ^aA^	24.92 ± 0.01 ^aA^	24.92 ± 0.01 ^aA^	24.92 ± 0.01 ^aA^
	2 h	24.80 ± 0.01 ^aA^	24.83 ± 0.01 ^aA^	24.84 ± 0.01 ^aA^	24.67 ± 0.01 ^aA^	24.63 ± 0.01 ^aA^
	4 h	23.31 ± 0.03 ^bA^	23.37 ± 0.02 ^bA^	23.41 ± 0.01 ^bA^	23.49 ± 0.02 ^bA^	23.52 ± 0.01 ^bA^
	8 h	20.63 ± 0.02 ^cB^	20.72 ± 0.01 ^cB^	20.77 ± 0.02 ^cB^	22.79 ± 0.02 ^cA^	21.88 ± 0.02 ^cA^
OB3	0 h	27.68 ± 0.00 ^aA^	27.68 ± 0.00 ^aA^	27.68 ± 0.00 ^aA^	27.68 ± 0.00 ^aA^	27.68 ± 0.00 ^aA^
	2 h	27.61 ± 0.01 ^aA^	27.65 ± 0.02 ^aA^	27.67 ± 0.02 ^aA^	27.68 ± 0.01 ^aA^	27.69 ± 0.01 ^aA^
	4 h	25.64 ± 0.03 ^bB^	25.68 ± 0.02 ^bB^	25.71 ± 0.01 ^bB^	26.15 ± 0.02 ^bA^	26.41 ± 0.02 ^bA^
	8 h	23.92 ± 0.02 ^cB^	23.97 ± 0.02 ^cB^	24.00 ± 0.02 ^cB^	25.70 ± 0.03 ^cA^	25.82 ± 0.03 ^cA^

Values are expressed as mean ± standard deviation (*n* = 3) and reported as % total FAMEs. Different lowercase superscript letters indicate significant differences among frying times within the same oil blend and essential oil concentration. Different uppercase superscript letters indicate significant differences among essential oil concentrations at the same frying time within the same oil blend (two-way ANOVA followed by Tukey’s post hoc test, *p* < 0.05). 0 h: unheated oil before frying (initial state prior to thermal treatment).

**Table 4:** Changes in n-3 fatty acid content of vegetable oil blends during deep-frying with cinnamon essential oil.

Oil Blend	Time	0 ppm	200 ppm	400 ppm	800 ppm	1200 ppm
OB1	0 h	16.64 ± 0.01 ^aA^	16.64 ± 0.01 ^aA^	16.64 ± 0.01 ^aA^	16.64 ± 0.01 ^aA^	16.64 ± 0.01 ^aA^
	2 h	16.54 ± 0.02 ^aA^	16.55 ± 0.01 ^aA^	16.60 ± 0.01 ^aA^	16.54 ± 0.01 ^aA^	16.49 ± 0.01 ^aA^
	4 h	14.05 ± 0.01 ^bA^	14.05 ± 0.02 ^bA^	14.07 ± 0.01 ^bA^	14.08 ± 0.02 ^bB^	14.08 ± 0.01 ^bA^
	8 h	10.01 ± 0.01 ^cC^	10.02 ± 0.00 ^cC^	10.08 ± 0.02 ^cB^	12.17 ± 0.02 ^cA^	12.25 ± 0.02 ^cA^
OB2	0 h	8.42 ± 0.02 ^aA^	8.42 ± 0.02 ^aA^	8.42 ± 0.02 ^aA^	8.42 ± 0.02 ^aA^	8.42 ± 0.02 ^aA^
	2 h	8.34 ± 0.04 ^aA^	8.36 ± 0.03 ^aA^	8.38 ± 0.03 ^aA^	8.35 ± 0.02 ^aA^	8.38 ± 0.02 ^aA^
	4 h	6.25 ± 0.02 ^bA^	6.26 ± 0.03 ^bA^	6.29 ± 0.02 ^bB^	6.32 ± 0.03 ^bC^	6.33 ± 0.02 ^bB^
	8 h	2.91 ± 0.02 ^cB^	2.95 ± 0.03 ^cB^	2.98 ± 0.03 ^cB^	5.17 ± 0.04 ^cA^	5.27 ± 0.04 ^cA^
OB3	0 h	5.52 ± 0.01 ^aA^	5.52 ± 0.01 ^aA^	5.52 ± 0.01 ^aA^	5.52 ± 0.01 ^aA^	5.52 ± 0.01 ^aA^
	2 h	5.42 ± 0.03 ^aA^	5.45 ± 0.02 ^aA^	5.49 ± 0.02 ^aA^	5.47 ± 0.01 ^aA^	5.46 ± 0.01 ^aA^
	4 h	2.40 ± 0.01 ^bA^	2.42 ± 0.02 ^bA^	2.47 ± 0.01 ^bB^	3.25 ± 0.02 ^bA^	3.26 ± 0.02 ^bB^
	8 h	0.01 ± 0.00 ^cB^	0.01 ± 0.00 ^cB^	0.02 ± 0.00 ^cB^	1.29 ± 0.03 ^cA^	1.35 ± 0.03 ^cA^

Values are expressed as mean ± standard deviation (*n* = 3) and reported as % total FAMEs. Different lowercase superscript letters indicate significant differences among frying times within the same oil blend and essential oil concentration. Different uppercase superscript letters indicate significant differences among essential oil concentrations at the same frying time within the same oil blend (two-way ANOVA followed by Tukey’s post hoc test, *p* < 0.05). 0 h: unheated oil before frying (initial state prior to thermal treatment).

**Table 5:** The oxidative stability of new oil blends under deep frying conditions.

Contents	Oil Type	Fresh OB	Repeated Deep Frying			
			2 h	4 h	6 h	8 h
PV (Meq O_2_/kg)	OB1	1.22 ^eB^ ± 0.03	2.14 ^dC^ ± 0.11	5.89 ^cA^ ± 0.05	8.23 ^bA^ ± 0.09	9.14 ^aA^ ± 0.10
	OB2	1.29 ^eA^ ± 0.01	3.14 ^dB^ ± 0.09	5.1 ^cC^ ± 0.06	7.34 ^bB^ ± 0.05	9.06 ^aA^ ± 0.06
	OB3	1.21 ^eA^ ± 0.00	3.62 ^dA^ ± 0.10	5.32 ^cB^ ± 0.04	7.76 ^bC^ ± 0.08	8.87 ^aB^ ± 0.05
AV (mg KOH/g)	OB1	0.15 ^eA^ ± 0.00	0.21 ^dB^ ± 0.02	0.31 ^cA^ ± 0.02	0.68 ^bA^ ± 0.09	0.95 ^aA^ ± 0.05
	OB2	0.17 ^dB^ ± 0.01	0.26 ^dA^ ± 0.02	0.37 ^cA^ ± 0.06	0.62 ^bA^ ± 0.09	0.83 ^aB^ ± 0.06
	OB3	0.16 ^dAB^ ± 0.02	0.23 ^cB^ ± 0.04	0.27 ^cB^ ± 0.01	0.53 ^bB^ ± 0.00	0.71 ^aC^ ± 0.03
MDA (mg/kg)	OB1	0.02 ^dA^ ± 0.00	0.05 ^dA^ ± 0.01	0.12 ^cA^ ± 0.03	0.48 ^bA^ ± 0.03	0.72 ^aA^ ± 0.04
	OB2	0.01 ^dA^ ± 0.00	0.02 ^dB^ ± 0.02	0.12 ^cA^ ± 0.01	0.18 ^bB^ ± 0.02	0.42 ^aB^ ± 0.04
	OB3	0.01 ^cA^ ± 0.01	0.03 ^dB^ ± 002	0.09 ^cB^ ± 0.02	0.19 ^bB^ ± 0.05	0.42 ^aB^ ± 0.06
AnV (Unit)	OB1	2.02 ^eB^ ± 0.01	5.16 ^dB^ ± 0.15	25.14 ^cA^ ± 0.10	48.21 ^bA^ ± 0.81	64.89 ^aA^ ± 0.78
	OB2	2.11 ^eA^ ± 0.01	6.13 ^dA^ ± 0.08	20.21 ^cB^ ± 0.08	45.66 ^bB^ ± 0.06	64.22 ^aA^ ± 0.98
	OB3	2.06 ^eC^ ± 0.01	6.13 ^dA^ ± 0.02	17.07 ^cC^ ± 0.07	36.87 ^bC^ ± 0.11	54.14 ^aB^ ± 0.64

Values are expressed as mean ± standard deviation (*n* = 3). Different lowercase superscript letters indicate significant differences among frying times within the same oil blend. Different uppercase superscript letters indicate significant differences among oil blends at the same frying time (two-way ANOVA followed by Tukey’s post hoc test, *p* < 0.05).

**Table 6:** Kinetic parameters of lipid oxidation indices during heating treatment.

Sample	Model	k	R^2^	*p*-Value	RMSE
OB1-MDA	Zero-order	0.0915 ± 0.0193	0.882	0.0179	0.122
OB2-MDA	Zero-order	0.0490 ± 0.0112	0.864	0.0223	0.071
OB3-MDA	Zero-order	0.049 ± 0.0116	0.857	0.0241	0.073
OB1-PV	First-order	0.2688 ± 0.0468	0.917	0.0105	0.296
OB2-PV	First-order	0.2376 ± 0.0370	0.932	0.0076	0.234
OB3-PV	First-order	0.2373 ± 0.0501	0.882	0.0178	0.317

Note: k values are presented as mean ± standard error. Zero-order and first-order kinetic models were applied for MDA and PV changes, respectively.

**Table 7:** Effect of essential oils on peroxide value (PV, Meq O_2_/kg) of vegetable oil blends during deep-frying.

Oil Blend	Essential Oil	Time	0 ppm	200 ppm	400 ppm	800 ppm	1200 ppm
OB1	Basil	0 h	1.22 ± 0.03 ^dA^	1.22 ± 0.00 ^dA^	1.22 ± 0.00 ^dA^	1.22 ± 0.00 ^cA^	1.22 ± 0.00 ^dA^
		2 h	2.14 ± 0.11 ^cD^	2.32 ± 0.01 ^cD^	2.12 ± 0.02 ^cC^	2.06 ± 0.01 ^cA^	2.02 ± 0.00 ^cB^
		4 h	5.89 ± 0.05 ^bD^	5.78 ± 0.02 ^bD^	4.67 ± 0.02 ^bC^	4.21 ± 0.02 ^bA^	4.15 ± 0.00 ^bB^
		8 h	9.14 ± 0.10 ^aA^	7.43 ± 0.01 ^aA^	7.23 ± 0.02 ^aB^	6.91 ± 0.02 ^aC^	6.34 ± 0.02 ^aD^
OB2	Basil	0 h	1.29 ± 0.01 ^aA^	1.29 ± 0.01 ^aA^	1.29 ± 0.01 ^dA^	1.29 ± 0.01 ^dA^	1.29 ± 0.01 ^dA^
		2 h	3.14 ± 0.09 ^bD^	2.94 ± 0.00 ^bD^	2.34 ± 0.00 ^cC^	2.40 ± 0.00 ^cA^	2.32 ± 0.01 ^cB^
		4 h	5.10 ± 0.06 ^cD^	5.02 ± 0.00 ^cD^	4.98 ± 0.02 ^bC^	4.29 ± 0.01 ^bA^	4.23 ± 0.03 ^bB^
		8 h	9.06 ± 0.06 ^dA^	8.32 ± 0.02 ^dA^	7.62 ± 0.00 ^aB^	7.01 ± 0.01 ^aC^	6.32 ± 0.01 ^bD^
OB3	Basil	0 h	1.21 ± 0.00 ^dA^	1.21 ± 0.00 ^dA^	1.21 ± 0.00 ^dA^	1.21 ± 0.00 ^dA^	1.21 ± 0.00 ^dA^
		2 h	3.62 ± 0.10 ^cA^	3.10 ± 0.00 ^cA^	3.08 ± 0.00 ^cA^	2.97 ± 0.01 ^cB^	2.91 ± 0.02 ^aB^
		4 h	5.32 ± 0.04 ^bA^	5.43 ± 0.02 ^bA^	4.53 ± 0.00 ^bB^	4.23 ± 0.00 ^bC^	4.32 ± 0.01 ^bC^
		8 h	8.87 ± 0.05 ^aA^	7.65 ± 0.01 ^aA^	7.45 ± 0.01 ^aB^	6.95 ± 0.03 ^aC^	6.62 ± 0.02 ^aC^
OB1	Cinnamon	0 h	1.02 ± 0.00 ^dA^	1.02 ± 0.00 ^dA^	1.02 ± 0.00 ^dA^	1.02 ± 0.00 ^dA^	1.02 ± 0.00 ^dA^
		2 h	2.31 ± 0.02 ^cA^	2.12 ± 0.01 ^cA^	2.02 ± 0.00 ^cB^	2.00 ± 0.01 ^cB^	2.00 ± 0.01 ^cB^
		4 h	5.58 ± 0.03 ^bA^	5.45 ± 0.01 ^bA^	4.54 ± 0.02 ^bB^	4.26 ± 0.02 ^bC^	4.10 ± 0.02 ^bD^
		8 h	7.31 ± 0.01 ^aA^	7.03 ± 0.02 ^aA^	6.62 ± 0.03 ^aB^	6.32 ± 0.00 ^aC^	5.56 ± 0.03 ^aD^
OB2	Cinnamon	0 h	1.29 ± 0.00 ^dA^	1.29 ± 0.00 ^dA^	1.29 ± 0.00 ^dA^	1.29 ± 0.00 ^dA^	1.29 ± 0.00 ^dA^
		2 h	2.93 ± 0.01 ^cA^	2.84 ± 0.00 ^cA^	2.71 ± 0.02 ^cB^	2.70 ± 0.03 ^cC^	2.65 ± 0.00 ^cD^
		4 h	5.61 ± 0.03 ^bA^	5.43 ± 0.00 ^bA^	4.57 ± 0.03 ^bB^	4.35 ± 0.01 ^bC^	4.03 ± 0.03 ^bD^
		8 h	8.35 ± 0.05 ^aA^	8.02 ± 0.00 ^aA^	6.54 ± 0.01 ^dB^	6.21 ± 0.02 ^aC^	5.76 ± 0.04 ^aD^
OB3	Cinnamon	0 h	1.21 ± 0.01 ^dA^	1.21 ± 0.01 ^dA^	1.21 ± 0.01 ^dA^	1.21 ± 0.01 ^dA^	1.21 ± 0.01 ^dA^
		2 h	3.23 ± 0.03 ^cA^	3.12 ± 0.02 ^cA^	2.96 ± 0.01 ^cB^	2.83 ± 0.05 ^cB^	2.71 ± 0.01 ^cB^
		4 h	5.21 ± 0.04 ^bA^	5.13 ± 0.01 ^bA^	4.46 ± 0.04 ^bB^	4.13 ± 0.02 ^bC^	3.92 ± 0.02 ^bD^
		8 h	7.78 ± 0.06 ^aA^	7.66 ± 0.03 ^aA^	6.76 ± 0.02 ^aB^	6.41 ± 0.01 ^aC^	5.78 ± 0.03 ^aD^

Values are expressed as mean ± standard deviation (*n* = 3). Different lowercase superscript letters indicate significant differences among frying times within the same oil blend and essential oil concentration. Different uppercase superscript letters indicate significant differences among essential oil concentrations at the same frying time within the same oil blend (two-way ANOVA followed by Tukey’s post hoc test, *p* < 0.05). 0 h: unheated oil before frying (initial state prior to thermal treatment).

**Table 8:** Effect of essential oils on acid value (AV, mg KOH/g) of vegetable oil blends during deep-frying.

Oil Blend	Essential Oil	Time	0 ppm	200 ppm	400 ppm	800 ppm	1200 ppm
OB1	Basil	0 h	0.15 ± 0.00 ^dA^	0.15 ± 0.02 ^dA^	0.15 ± 0.02 ^dA^	0.15 ± 0.02 ^cA^	0.15 ± 0.02 ^cA^
		2 h	0.21 ± 0.02 ^cA^	0.22 ± 0.01 ^cA^	0.21 ± 0.00 ^cA^	0.21 ± 0.00 ^cA^	0.20 ± 0.00 ^bA^
		4 h	0.31 ± 0.02 ^bA^	0.32 ± 0.00 ^bA^	0.26 ± 0.02 ^bB^	0.22 ± 0.00 ^bC^	0.21 ± 0.02 ^bC^
		8 h	0.95 ± 0.05 ^aA^	0.63 ± 0.02 ^aA^	0.61 ± 0.00 ^aB^	0.57 ± 0.00 ^aC^	0.43 ± 0.01 ^aD^
OB2	Basil	0 h	0.17 ± 0.01 ^dA^	0.17 ± 0.00 ^dA^	0.17 ± 0.00 ^cA^	0.17 ± 0.00 ^cA^	0.17 ± 0.00 ^cA^
		2 h	0.26 ± 0.02 ^cA^	0.23 ± 0.02 ^cA^	0.20 ± 0.01 ^bB^	0.19 ± 0.02 ^cA^	0.19 ± 0.01 ^bA^
		4 h	0.37 ± 0.06 ^bA^	0.34 ± 0.01 ^bA^	0.25 ± 0.01 ^bB^	0.23 ± 0.00 ^bB^	0.23 ± 0.00 ^bB^
		8 h	0.83 ± 0.06 ^aA^	0.67 ± 0.00 ^aA^	0.51 ± 0.01 ^aB^	0.49 ± 0.01 ^aC^	0.44 ± 0.00 ^aD^
OB3	Basil	0 h	0.16 ± 0.02 ^dA^	0.16 ± 0.01 ^dA^	0.16 ± 0.01 ^dA^	0.16 ± 0.01 ^dA^	0.16 ± 0.01 ^dA^
		2 h	0.23 ± 0.04 ^dA^	0.22 ± 0.00 ^dA^	0.21 ± 0.00 ^cA^	0.21 ± 0.01 ^cA^	0.21 ± 0.02 ^cA^
		4 h	0.27 ± 0.01 ^bA^	0.32 ± 0.00 ^bA^	0.28 ± 0.00 ^bB^	0.25 ± 0.01 ^bC^	0.22 ± 0.00 ^bD^
		8 h	0.71 ± 0.03 ^aA^	0.61 ± 0.00 ^aA^	0.57 ± 0.01 ^aB^	0.48 ± 0.02 ^aC^	0.42 ± 0.01 ^aD^
OB1	Cinnamon	0 h	0.14 ± 0.01 ^dA^	0.14 ± 0.01 ^dA^	0.15 ± 0.01 ^cA^	0.15 ± 0.01 ^dA^	0.15 ± 0.01 ^dA^
		2 h	0.28 ± 0.01 ^cC^	0.21 ± 0.00 ^cC^	0.23 ± 0.00 ^bB^	0.20 ± 0.00 ^bA^	0.21 ± 0.00 ^cA^
		4 h	0.29 ± 0.00 ^bA^	0.22 ± 0.01 ^bA^	0.22 ± 0.00 ^bA^	0.19 ± 0.00 ^cB^	0.18 ± 0.00 ^bB^
		8 h	0.69 ± 0.02 ^aA^	0.65 ± 0.00 ^aA^	0.53 ± 0.00 ^aB^	0.45 ± 0.01 ^aC^	0.34 ± 0.01 ^aD^
OB2	Cinnamon	0 h	0.17 ± 0.01 ^dA^	0.17 ± 0.00 ^dA^	0.17 ± 0.00 ^cA^	0.17 ± 0.00 ^cA^	0.17 ± 0.00 ^cA^
		2 h	0.26 ± 0.01 ^cA^	0.22 ± 0.01 ^cA^	0.21 ± 0.00 ^bA^	0.20 ± 0.00 ^bA^	0.19 ± 0.00 ^bA^
		4 h	0.39 ± 0.01 ^bA^	0.32 ± 0.01 ^bA^	0.23 ± 0.01 ^bB^	0.22 ± 0.01 ^bB^	0.20 ± 0.00 ^bB^
		8 h	0.80 ± 0.02 ^aA^	0.71 ± 0.02 ^aA^	0.54 ± 0.01 ^aB^	0.47 ± 0.00 ^aC^	0.36 ± 0.01 ^aD^
OB3	Cinnamon	0 h	0.15 ± 0.01 ^dA^	0.15 ± 0.01 ^dA^	0.15 ± 0.01 ^dA^	0.15 ± 0.01 ^dA^	0.15 ± 0.01 ^dA^
		2 h	0.26 ± 0.01 ^cA^	0.22 ± 0.00 ^cA^	0.21 ± 0.00 ^cB^	0.20 ± 0.00 ^cB^	0.19 ± 0.00 ^cB^
		4 h	0.38 ± 0.02 ^bA^	0.30 ± 0.01 ^bA^	0.25 ± 0.00 ^bB^	0.23 ± 0.00 ^bB^	0.22 ± 0.00 ^bB^
		8 h	0.69 ± 0.02 ^aA^	0.66 ± 0.01 ^aA^	0.51 ± 0.02 ^aB^	0.45 ± 0.01 ^aC^	0.42 ± 0.01 ^aC^

Values are expressed as mean ± standard deviation (*n* = 3). Different lowercase superscript letters indicate significant differences among frying times within the same oil blend and essential oil concentration. Different uppercase superscript letters indicate significant differences among essential oil concentrations at the same frying time within the same oil blend (two-way ANOVA followed by Tukey’s post hoc test, *p* < 0.05). 0 h: unheated oil before frying (initial state prior to thermal treatment).

**Table 9:** Effect of essential oils on malondialdehyde content (MDA, mg/kg) of vegetable oil blends during deep-frying.

Oil Blend	Essential Oil	Time	0 ppm	200 ppm	400 ppm	800 ppm	1200 ppm
OB1	Basil	0 h	0.02 ± 0.00 ^cA^	0.02 ± 0.00 ^cA^	0.02 ± 0.00 ^cA^	0.02 ± 0.00 ^cA^	0.02 ± 0.00 ^cA^
		2 h	0.05 ± 0.01 ^cA^	0.04 ± 0.01 ^cA^	0.03 ± 0.00 ^cD^	0.06 ± 0.00 ^cC^	0.03 ± 0.00 ^cA^
		4 h	0.12 ± 0.03 ^bA^	0.11 ± 0.01 ^bA^	0.12 ± 0.00 ^bA^	0.12 ± 0.01 ^bA^	0.11 ± 0.01 ^bA^
		8 h	0.72 ± 0.04 ^cA^	0.38 ± 0.01 ^cA^	0.37 ± 0.00 ^aA^	0.40 ± 0.01 ^aA^	0.38 ± 0.01 ^aA^
OB2	Basil	0 h	0.01 ± 0.00 ^cA^	0.01 ± 0.00 ^cA^	0.02 ± 0.00 ^dA^	0.01 ± 0.00 ^dA^	0.01 ± 0.00 ^dA^
		2 h	0.02 ± 0.02 ^cB^	0.02 ± 0.00 ^cB^	0.12 ± 0.00 ^cA^	0.10± 0.00 ^cA^	0.04 ± 0.00 ^cB^
		4 h	0.12 ± 0.01 ^bC^	0.12 ± 0.00 ^bC^	0.40 ± 0.00 ^aA^	0.21 ± 0.00 ^bB^	0.12 ± 0.00 ^bC^
		8 h	0.42 ± 0.04 ^aA^	0.41 ± 0.01 ^aA^	0.36 ± 0.02 ^bB^	0.35 ± 0.02 ^aB^	0.32 ± 0.02 ^aC^
OB3	Basil	0 h	0.01 ± 0.01 ^cA^	0.01 ± 0.00 ^cA^	0.01 ± 0.00 ^cA^	0.01 ± 0.00 ^dA^	0.01 ± 0.00 ^dA^
		2 h	0.03 ± 0.02 ^cA^	0.03 ± 0.00 ^cA^	0.03 ± 0.01 ^cA^	0.03 ± 0.02 ^cA^	0.04 ± 0.02 ^cA^
		4 h	0.09 ± 0.02 ^bA^	0.12 ± 0.02 ^bA^	0.11 ± 0.02 ^bA^	0.11 ± 0.01 ^bA^	0.12 ± 0.01 ^bA^
		8 h	0.42 ± 0.06 ^aA^	0.43 ± 0.01 ^aA^	0.42 ± 0.00 ^aA^	0.40 ± 0.01 ^aA^	0.34 ± 0.01 ^aB^
OB1	Cinnamon	0 h	0.02 ± 0.00 ^cA^	0.02 ± 0.00 ^cA^	0.02 ± 0.00 ^cA^	0.02 ± 0.00 ^cA^	0.02 ± 0.00 ^cA^
		2 h	0.05 ± 0.00 ^cA^	0.03 ± 0.01 ^cA^	0.02 ± 0.00 ^cA^	0.01 ± 0.00 ^cA^	0.02 ± 0.00 ^cA^
		4 h	0.31 ± 0.01 ^bA^	0.29 ± 0.01 ^bA^	0.17 ± 0.00 ^bB^	0.11 ± 0.01 ^bC^	0.06 ± 0.01 ^bD^
		8 h	0.46 ± 0.02 ^aA^	0.41 ± 0.01 ^aA^	0.35 ± 0.00 ^aB^	0.30 ± 0.01 ^aC^	0.31 ± 0.01 ^aC^
OB2	Cinnamon	0 h	0.01 ± 0.00 ^cA^	0.01 ± 0.00 ^cA^	0.01 ± 0.00 ^cA^	0.01 ± 0.00 ^cA^	0.01 ± 0.00 ^cdA^
		2 h	0.03 ± 0.00 ^cA^	0.02 ± 0.00 ^cA^	0.03 ± 0.00 ^cA^	0.02± 0.00 ^cA^	0.02 ± 0.00 ^bcA^
		4 h	0.11 ± 0.01 ^bA^	0.08 ± 0.00 ^bA^	0.08 ± 0.00 ^bA^	0.06 ± 0.00 ^bA^	0.05 ± 0.00 ^bA^
		8 h	0.48 ± 0.02 ^aA^	0.44 ± 0.01 ^aA^	0.36 ± 0.02 ^aB^	0.35 ± 0.02 ^aB^	0.30 ± 0.02 ^aC^
OB3	Cinnamon	0 h	0.01 ± 0.00 ^cA^	0.01 ± 0.00 ^cA^	0.01 ± 0.00 ^cA^	0.01 ± 0.00 ^cA^	0.01 ± 0.00 ^cA^
		2 h	0.05 ± 0.01 ^cA^	0.03 ± 0.00 ^cA^	0.02 ± 0.01 ^cA^	0.02 ± 0.02 ^cA^	0.02 ± 0.02 ^cA^
		4 h	0.14 ± 0.01 ^bAB^	0.10 ± 0.02 ^bAB^	0.12 ± 0.02 ^bA^	0.09 ± 0.01 ^bB^	0.07 ± 0.01 ^bB^
		8 h	0.49 ± 0.01 ^aA^	0.46 ± 0.01 ^aA^	0.39 ± 0.00 ^aB^	0.32 ± 0.01 ^aC^	0.30 ± 0.01 ^aC^

Values are expressed as mean ± standard deviation (*n* = 3). Different lowercase superscript letters indicate significant differences among frying times within the same oil blend and essential oil concentration. Different uppercase superscript letters indicate significant differences among essential oil concentrations at the same frying time within the same oil blend (two-way ANOVA followed by Tukey’s post hoc test, *p* < 0.05). 0 h: unheated oil before frying (initial state prior to thermal treatment).

**Table 10:** Effect of essential oils on anisidine value (AnV) of vegetable oil blends during deep-frying.

Oil Blend	Essential Oil	Time	0 ppm	200 ppm	400 ppm	800 ppm	1200 ppm
OB1	Basil	0 h	2.02 ± 0.00 ^dA^	2.02 ± 0.00 ^dA^	2.02 ± 0.00 ^dA^	2.02 ± 0.00 ^dA^	2.02 ± 0.00 ^dA^
		2 h	0.05 ± 0.01 ^cA^	5.42 ± 0.02 ^cA^	5.42 ± 0.03 ^cA^	5.15 ± 0.05 ^cA^	5.12 ± 0.01 ^cA^
		4 h	0.12 ± 0.03 ^bA^	18.03 ± 0.03 ^bA^	16.13 ± 0.01 ^bB^	16.10 ± 0.03 ^bB^	15.13 ± 0.01 ^bB^
		8 h	0.72 ± 0.04 ^aA^	45.43 ± 0.02 ^aA^	42.32 ± 0.01 ^aB^	39.15 ± 0.03 ^aC^	38.13 ± 0.03 ^aC^
OB2	Basil	0 h	2.11 ± 0.00 ^dA^	2.11 ± 0.01 ^dA^	2.11 ± 0.01 ^dA^	2.11 ± 0.01 ^dA^	2.11 ± 0.01 ^dA^
		2 h	0.02 ± 0.02 ^cA^	5.87 ± 0.01 ^cA^	5.85 ± 0.01 ^cA^	5.35 ± 0.03 ^cA^	5.33 ± 0.01 ^cA^
		4 h	0.12 ± 0.01 ^bA^	19.07 ± 0.00 ^bA^	17.12 ± 0.03 ^bB^	17.11 ± 0.03 ^bB^	16.34 ± 0.03 ^bB^
		8 h	0.42 ± 0.04 ^aA^	42.46 ± 0.00 ^aA^	43.23 ± 0.02 ^aA^	38.69 ± 0.02 ^aB^	37.56 ± 0.02 ^aB^
OB3	Basil	0 h	2.06 ± 0.01 ^dA^	2.06 ± 0.01 ^dA^	2.06 ± 0.01 ^dA^	2.06 ± 0.01 ^dA^	2.06 ± 0.01 ^dA^
		2 h	0.03 ± 0.02 ^cA^	6.33 ± 0.03 ^cA^	6.13 ± 0.02 ^cA^	6.15 ± 0.04 ^cA^	6.12 ± 0.00 ^cA^
		4 h	0.09 ± 0.02 ^bA^	18.46 ± 0.02 ^bA^	17.23 ± 0.04 ^bB^	17.33 ± 0.01 ^bB^	17.16 ± 0.03 ^bC^
		8 h	0.42 ± 0.06 ^aA^	43.78 ± 0.01 ^aA^	41.45 ± 0.00 ^aB^	39.31 ± 0.03 ^aC^	36.23 ± 0.05 ^aD^
OB1	Cinnamon	0 h	2.02 ± 0.01 ^dA^	2.02 ± 0.01 ^dA^	2.02 ± 0.01 ^dA^	2.02 ± 0.01 ^dA^	2.02 ± 0.01 ^dA^
		2 h	5.41 ± 0.01 ^cA^	5.34 ± 0.00 ^cA^	5.22 ± 0.01 ^cA^	5.00 ± 0.02 ^cA^	5.01 ± 0.01 ^cA^
		4 h	18.12 ± 0.02 ^bA^	18.00 ± 0.03 ^bA^	15.14 ± 0.02 ^bB^	14.51 ± 0.01 ^bC^	14.45 ± 0.02 ^bB^
		8 h	45.63 ± 0.03 ^aA^	45.51 ± 0.02 ^aA^	36.65 ± 0.01 ^aB^	35.34 ± 0.02 ^aB^	30.32 ± 0.01 ^aC^
OB2	Cinnamon	0 h	2.11 ± 0.01 ^dA^	2.11 ± 0.01 ^dA^	2.11 ± 0.01 ^dA^	2.11 ± 0.01 ^dA^	2.11 ± 0.01 ^dA^
		2 h	5.36 ± 0.02 ^cA^	5.34 ± 0.01 ^cA^	5.34 ± 0.01 ^cA^	5.30 ± 0.01 ^bA^	5.23 ± 0.01 ^dA^
		4 h	19.15 ± 0.01 ^bA^	19.02 ± 0.02 ^bA^	19.02 ± 0.02 ^bA^	15.19 ± 0.01 ^bB^	15.02 ± 0.01 ^cB^
		8 h	42.52 ± 0.03 ^aA^	42.41 ± 0.02 ^aA^	36.65 ± 0.02 ^aB^	34.17 ± 0.02 ^cC^	32.12 ± 0.02 ^bD^
OB3	Cinnamon	0 h	2.06 ± 0.01 ^dA^	2.06 ± 0.01 ^dA^	2.06 ± 0.01 ^dA^	2.06 ± 0.01 ^dA^	2.06 ± 0.01 ^dA^
		2 h	6.79 ± 0.01 ^cA^	6.76 ± 0.00 ^cA^	6.53 ± 0.00 ^cB^	5.80 ± 0.01 ^cB^	5.79 ± 0.01 ^cB^
		4 h	18.23 ± 0.04 ^bA^	18.12 ± 0.02 ^bA^	14.83 ± 0.01 ^bB^	14.62 ± 0.00 ^bB^	14.05 ± 0.01 ^bB^
		8 h	43.75 ± 0.05 ^aA^	43.34 ± 0.01 ^aA^	36.24 ± 0.01 ^aB^	35.20 ± 0.03 ^aC^	33.12 ± 0.01 ^aD^

Values are expressed as mean ± standard deviation (*n* = 3). Different lowercase superscript letters indicate significant differences among frying times within the same oil blend and essential oil concentration. Different uppercase superscript letters indicate significant differences among essential oil concentrations at the same frying time within the same oil blend (two-way ANOVA followed by Tukey’s post hoc test, *p* < 0.05). 0 h: unheated oil before frying (initial state prior to thermal treatment).

## Data Availability

The original contributions presented in this study are included in the article/Supplementary Materials. Further inquiries can be directed to the corresponding author.

## Supplementary Materials

The following supporting information can be downloaded at: https://www.mdpi.com/article/10.3390/foods15132284/s1, Tabel S1: Baseline fatty acid composition of sunflower oil (SFO), flaxseed oil (FSO), olive oil (OVO), and palm kernel oil (PKO) used for oil blend formulation; Table S2: Changes in the n-6/n-3 fatty acid ratio calculated from fatty acid composition data (% total FAMEs) of vegetable oil blends during deep-frying.
