# Supplementary material for: Basil and Cinnamon Essential Oils Improve Oxidative Stability and Fatty Acid Composition of Vegetable Oil Blends During Deep-Frying
Source: Foods. 2026 Jun 25;15(13):2284. doi: 10.3390/foods15132284 (PMC13362435; doi:10.3390/foods15132284)
Supplement: Supplementary file 1 [file foods-15-02284-s001.zip › Supplementary Table S1.pdf]

Supplementary Table S1. Baseline fatty acid composition of sunflower oil (SFO), flaxseed oil (FSO), olive oil (OVO), and palm kernel oil (PKO) used for oil blend formulation

Fatty acid	SFO	FSO	OVO	PKO
C12:0	$0.10^{a} \pm 0.01$	$0.01^{b} \pm 0.01$	$0.02^{b} \pm 0.01$	$47.91^{c} \pm 0.02$
C14:0	$0.21^{a} \pm 0.01$	$0.00^{b} \pm 0.01$	$0.0^{b} \pm 0.02$	$16.03^{c} \pm 0.08$
C16:0	$7.72^{a} \pm 0.03$	$4.90^{b} \pm 0.02$	$10.67^{c} \pm 0.01$	$11.53^{d} \pm 0.03$
C16:1	$0.58^{a} \pm 0.02$	$0.00^{b} \pm 0.00$	$0.70^{c} \pm 0.03$	$0.01^{b} \pm 0.00$
C17:0	$0.77^{a} \pm 0.01$	$0.00^{b} \pm 0.00$	$0.02^{c} \pm 0.01$	$0.00^{d} \pm 0.00$
C18:0	$4.18^{a} \pm 0.10$	$3.40^{b} \pm 0.04$	$3.70^{c} \pm 0.05$	$4.66^{d} \pm 0.03$
C18:1	$22.25^{a} \pm 0.03$	$19.30^{b} \pm 0.04$	$78.91^{c} \pm 0.02$	$16.43^{d} \pm 0.04$
C18:2	$59.87^{a} \pm 0.08$	$14.80^{b} \pm 0.02$	$5.10^{c} \pm 0.01$	$2.70^{d} \pm 0.02$
C20:0	$0.43^{a} \pm 0.00$	$0.10^{b} \pm 0.02$	$0.05^{c} \pm 0.01$	$0.10^{b} \pm 0.00$
C18:3	$0.03^{a} \pm 0.00$	$57.27^{b} \pm 0.03$	$0.50^{c} \pm 0.03$	$0.10^{a} \pm 0.01$
C20:1	$2.98^{a} \pm 0.02$	$0.20^{b} \pm 0.00$	$0.20^{b} \pm 0.01$	$0.04^{c} \pm 0.00$
C22:0	$0.88^{a} \pm 0.02$	$0.02^{b} \pm 0.00$	$0.10^{c} \pm 0.01$	$0.49^{d} \pm 0.01$
**SFA**	**14.29**	**8.44**	**14.59**	**80.72**
**MUFA**	**25.81**	**19.50**	**79.81**	**16.48**
**PUFA**	**59.90**	**72.07**	**5.60**	**2.80**
**SFA/MUFA/FUFA**	**1/1.8/4.2**	**1/2.3/8.5**	**2.6/14.3/1**	**28.8/5.9/1**
**n-6/ n- 3**	**1995.6/1**	**0.3/1**	**10.2/1**	**27.0/1**

Each value in the table represents the mean ± standard deviations (n=3). Different lowercase letters in each line indicate a significant difference at $p < 0.05$.

C12:0 Lauric	C14:0 Myristic	C16:0 Palmitic	C16:1 Palmitoleic	SFA: Saturated fatty acid
C18:1 Oleic	C18:2 Linoleic	C18:3 Linolenic	C20: 0 Arachidic	MUFA: Monounsaturated fatty acid
SFO: Sunflower oil	FSO: Flaxseed oil	OVO: Olive oil	PKO: Palm kernel Oil	PUFA: Polyunsaturated fatty acid
