# Supplementary material for: Basil and Cinnamon Essential Oils Improve Oxidative Stability and Fatty Acid Composition of Vegetable Oil Blends During Deep-Frying
Source: Foods. 2026 Jun 25;15(13):2284. doi: 10.3390/foods15132284 (PMC13362435; doi:10.3390/foods15132284)
Supplement: Supplementary file 1 [file foods-15-02284-s001.zip › Supplementary Table S2.pdf]

Supplementary Table S2. Changes in the n-6/n-3 fatty acid ratio calculated from fatty acid composition data (% total FAMEs) of vegetable oil blends during deep-frying

Essential oil	Oil blend	Time	Control	200 ppm	400 ppm	800 ppm	1200 ppm
Basil EO	OB1	Fresh	1.00 ± 0.00^{aA}	–	–	–	–
		2 h	1.00 ± 0.00^{bB}	1.01 ± 0.00^{aA}	1.01 ± 0.00^{aA}	1.01 ± 0.00^{aA}	1.00 ± 0.00^{aA}
		4 h	1.06 ± 0.00^{cC}	1.07 ± 0.00^{bA}	1.06 ± 0.00^{bA}	1.27 ± 0.01^{bB}	1.07 ± 0.00^{bA}
		8 h	1.33 ± 0.01^{dD}	1.33 ± 0.01^{cA}	1.27 ± 0.01^{cB}	1.31 ± 0.01^{cC}	1.31 ± 0.01^{cC}
	OB2	Fresh	2.96 ± 0.01^{aA}	–	–	–	–
		2 h	2.96 ± 0.01^{bB}	2.96 ± 0.01^{aA}	2.97 ± 0.01^{aA}	2.99 ± 0.01^{aA}	2.99 ± 0.01^{aA}
		4 h	3.67 ± 0.02^{cC}	3.71 ± 0.02^{bA}	3.70 ± 0.01^{bA}	4.35 ± 0.02^{bB}	3.75 ± 0.02^{bA}
		8 h	6.59 ± 0.03^{dD}	6.90 ± 0.04^{cA}	4.35 ± 0.02^{cB}	5.27 ± 0.03^{cC}	5.27 ± 0.03^{cC}
	OB3	Fresh	5.01 ± 0.01^{aA}	–	–	–	–
		2 h	5.04 ± 0.01^{bB}	5.05 ± 0.02^{aA}	5.02 ± 0.02^{aA}	4.97 ± 0.01^{aA}	4.97 ± 0.01^{aA}
		4 h	7.70 ± 0.03^{cC}	10.45 ± 0.05^{bA}	10.29 ± 0.04^{bA}	8.88 ± 0.04^{bB}	10.72 ± 0.05^{bA}
		8 h	26.86 ± 0.11^{dD}	218.27 ± 1.25^{cA}	8.88 ± 0.04^{cB}	13.29 ± 0.08^{cC}	13.29 ± 0.08^{cC}
Cinnamon EO	OB1	Fresh	1.00 ± 0.00^{aA}	–	–	–	–
		2 h	0.97 ± 0.00^{bB}	1.00 ± 0.00^{aA}	1.01 ± 0.00^{aA}	1.00 ± 0.00^{aA}	1.00 ± 0.00^{aA}
		4 h	1.07 ± 0.01^{cC}	1.06 ± 0.01^{bA}	1.07 ± 0.01^{bA}	1.28 ± 0.01^{bB}	1.07 ± 0.01^{bA}
		8 h	1.34 ± 0.01^{dD}	1.34 ± 0.01^{cA}	1.28 ± 0.01^{cB}	1.31 ± 0.01^{cC}	1.31 ± 0.01^{cC}
	OB2	Fresh	2.96 ± 0.01^{aA}	–	–	–	–
		2 h	2.97 ± 0.01^{bB}	2.97 ± 0.01^{aA}	2.95 ± 0.01^{aA}	2.94 ± 0.01^{aA}	2.94 ± 0.01^{aA}
		4 h	3.73 ± 0.02^{cC}	3.72 ± 0.02^{bA}	3.70 ± 0.02^{bA}	4.41 ± 0.03^{bB}	3.73 ± 0.02^{bA}
		8 h	7.02 ± 0.03^{dD}	6.97 ± 0.04^{cA}	4.41 ± 0.03^{cB}	5.38 ± 0.03^{cC}	5.38 ± 0.03^{cC}
	OB3	Fresh	5.01 ± 0.01^{aA}	–	–	–	–
		2 h	5.07 ± 0.02^{bB}	5.04 ± 0.02^{aA}	5.05 ± 0.02^{aA}	5.10 ± 0.02^{aA}	5.10 ± 0.02^{aA}
		4 h	10.61 ± 0.05^{cC}	10.41 ± 0.05^{bA}	7.91 ± 0.04^{bB}	11.42 ± 0.06^{bC}	7.89 ± 0.04^{bB}
		8 h	>1000	>1000	11.42 ± 0.06^{cB}	20.08 ± 0.11^{cC}	20.08 ± 0.11^{cC}

Values are expressed as mean ± standard deviation (n = 3). Different lowercase superscript letters indicate significant differences among frying times within the same oil blend and essential oil concentration. Different uppercase superscript letters indicate significant differences among essential oil concentrations at the same frying time within the same oil blend (two-way ANOVA followed by Tukey's post hoc test, $p < 0.05$). Extremely high n-6/n-3 ratios (>1000) were observed due to near depletion of n-3 fatty acids during prolonged frying.
